# Post-Translational Modifications of BRD4: Therapeutic Targets for Tumor

**DOI:** 10.3389/fonc.2022.847701

**Published:** 2022-03-21

**Authors:** Na Liu, Rui Ling, Xiang Tang, Yunpeng Yu, Yuepeng Zhou, Deyu Chen

**Affiliations:** Institute of Oncology, Affiliated Hospital of Jiangsu University, Zhenjiang, China

**Keywords:** BRD4, post-translational modification, BET inhibitors, drug target, combination therapy

## Abstract

Bromodomain-containing protein 4 (BRD4), a member of the bromodomain and extraterminal (BET) family, is considered to be a major driver of cancer cell growth and a new target for cancer therapy. Over 30 targeted inhibitors currently in preclinical and clinical trials have significant inhibitory effects on various tumors, including acute myelogenous leukemia (AML), diffuse large B cell lymphoma, prostate cancer, breast cancer and so on. However, resistance frequently occurs, revealing the limitations of BET inhibitor (BETi) therapy and the complexity of the BRD4 expression mechanism and action pathway. Current studies believe that when the internal and external environmental conditions of cells change, tumor cells can directly modify proteins by posttranslational modifications (PTMs) without changing the original DNA sequence to change their functions, and epigenetic modifications can also be activated to form new heritable phenotypes in response to various environmental stresses. In fact, research is constantly being supplemented with regards to that the regulatory role of BRD4 in tumors is closely related to PTMs. At present, the PTMs of BRD4 mainly include ubiquitination and phosphorylation; the former mainly regulates the stability of the BRD4 protein and mediates BETi resistance, while the latter is related to the biological functions of BRD4, such as transcriptional regulation, cofactor recruitment, chromatin binding and so on. At the same time, other PTMs, such as hydroxylation, acetylation and methylation, also play various roles in BRD4 regulation. The diversity, complexity and reversibility of posttranslational modifications affect the structure, stability and biological function of the BRD4 protein and participate in the occurrence and development of tumors by regulating the expression of tumor-related genes and even become the core and undeniable mechanism. Therefore, targeting BRD4-related modification sites or enzymes may be an effective strategy for cancer prevention and treatment. This review summarizes the role of different BRD4 modification types, elucidates the pathogenesis in the corresponding cancers, provides a theoretical reference for identifying new targets and effective combination therapy strategies, and discusses the opportunities, barriers, and limitations of PTM-based therapies for future cancer treatment.

## Introduction

**Figure 1:**
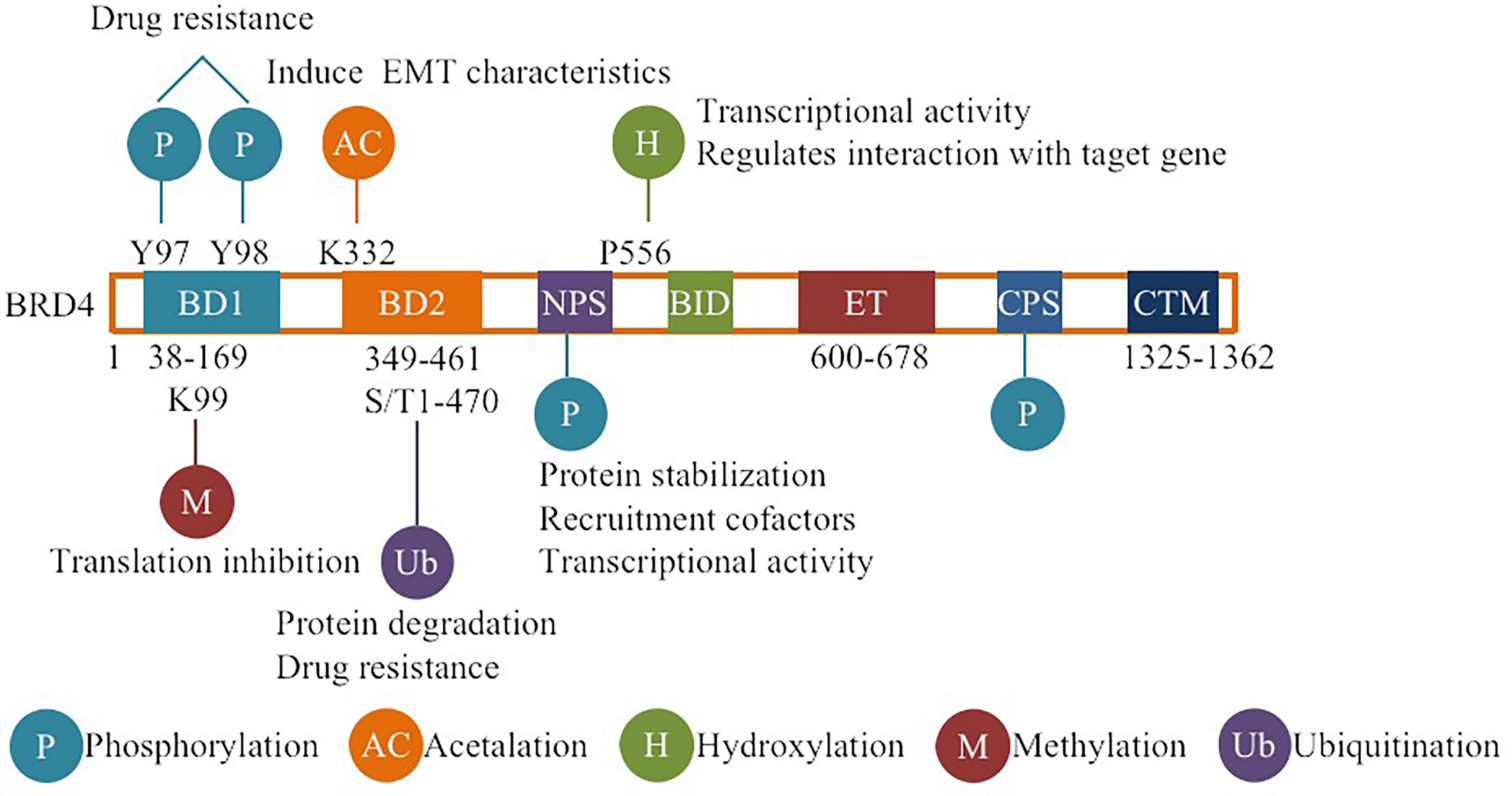
Overview of BRD4 PTMs: The major sites for BRD4 modifications (phosphorylation, ubiquitination, acetylation, methylation, hydroxylation) are plotted. Different colors are used to differentiate distinct modification types. The representative functions of some modifications are pointed out.

BRD4 is widely involved in normal cell function or body development and the occurrence and development of diseases such as fibrosis, chronic inflammation, viral infectious diseases and tumors under pathological conditions (1–6). Therefore, BRD4 is the most studied member of the BET family with two tandem N-terminal bromodomains (BD1 and BD2) and an extra C-terminal domain (ET), where BDs are used to recognize acetylated lysine residues and anchor to the corresponding sites, then acting as frame molecules that recruit transcriptional coactivators to promoters and superenhancers that drive downstream gene transcription to function (7, 8). BRD4 regulates downstream gene expression by combining its bromodomains with acetylated histone and lysine residues at histone H3 and H4 sites on chromatin and actively recruits positive transcription extension factor b (P-TEFb) to promote the transcriptional activation of RNA polymerase II (RNAPII) (9). In addition, BRD4 can regulate transcription initiation and extension by occupying the distal enhancer RNA (eRNA) region and can also recognize acetylated lysine residues on nonhistones (such as rela) and play a more complex and finer regulatory role (10, 11). In addition to regulating transcription, BRD4 also activates the repair and assembly of damaged DNA, promotes telomere extension, and maintains the self-renewal and pluripotency of embryonic stem cells (ESCs) (10, 12). Since BRD4 plays many important roles in cell life, its functional regulation is obviously crucial. Almost all proteins in cells undergo PTMs during their life cycle, and the self-expression and stabilization of proteins is believed to be mainly dependent on PTMs (13, 14). PTMs are a group of reversible protein modifications that affect the charge state, hydrophobicity, conformation or stability of a protein, and ultimately its function. The PTMs include the addition of functional groups, such as phosphorylation, acetylation (15, 16). Because proteins can be modified in different ways, they have different roles in different modification states. In addition, different sites with the same modification may have different effects on protein function. Therefore, the combination of different modifications and site changes lead to the diversity and complexity of protein functions (15, 17, 18). According to reports, multiple PTMs can also act in concert or compete for the same position (15, 17), for example, ubiquitination and acetylation of the tumor suppressor p53 can block each other at some specific sites, and phosphorylation can promote or inhibit acetylation, which is determined by different sites (19). Crosstalks among ubiquitination, phosphorylation, and acetylation of P53 greatly affect p53 protein stability and function, regulate p53-related signal transduction pathways, and participate in disease-related processes, especially cancer (19). PTMs of target proteins have been implicated in the regulation of tumorigenesis, suggesting that PTMs of target proteins are closely related to the development of cancer and suggesting the future of targeted PTM therapy (20, 21). At present, the PTMs of BRD4 mainly include ubiquitination, phosphorylation, methylation, hydroxylation, acetylation, etc. (**Figure 1**). The basic working mechanism of these modifications affects the stability, expression, localization and functional implementation of BRD4, causes protein conformational changes, and mediates the recruitment of binding partners and the activation of oncogenes. In addition, the basic working mechanism of these modifications also plays a signal transduction role in a series of processes, such as tumor occurrence and development and therapeutic drug resistance. This review introduces some typical examples of each modification type and discusses the therapeutic potential of targeting BRD4 modification.

## Ubiquitination

Ubiquitination and deubiquitination are common types of PTMs involving several different cellular processes, such as signal transduction, stress response, DNA repair, and apoptosis, thus, the disorder of these PTMs is involved in the occurrence and progression of cancer (22–26). Ubiquitination is the covalent binding of the polypeptide ubiquitin and target protein, which is completed by three enzymes: ubiquitin activating enzyme (E1), ubiquitin-conjugating enzyme (E2) and ubiquitin ligase (E3) (25). In short, E1 activates a single free ubiquitin, then E1 delivers the activated ubiquitin to E2, and finally E3 recruits a specific substrate and ubiquitin-conjugated E2 and mediates the transfer of ubiquitin from E2 to the target protein, the target protein modified by ubiquitination will be recognized by the proteasome and then degraded (25, 27). This process is reversed by deubiquitinase (DUB), which promotes protein stability by antagonizing E3 ubiquitin ligase-mediated protein polyubiquitination and proteasome degradation and removing the ubiquitin modification of target proteins (26). Dysregulation of ubiquitination and deubiquitination is often happened in tumors, and to regulate the formation and abundance of major signaling complexes and regulate tumor inhibition and tumor promotion signaling pathways in an environment-dependent manner (25, 28, 29). Therefore, a better understanding of the mechanism of ubiquitination regulation and function in tumorigenesis is conducive to the identification of new anticancer therapeutic targets. At present, targeted ubiquitin proteasome system (UPS) therapy has been proven to be effective in a variety of tumors (**Table 1**).

**Table 1:** Summary of targeted ubiquitin proteasome system therapy.

UPS	Inhibitor or compound	tumor	reference
Proteasome	Bortezomib	Multiple myeloma	(30)
		Acute lymphoblastic leukemia	(31)
E1 enzyme	TAK-243	Myeloma	(32)
	Pevonedistat (MLN4924)	Colorectal cancer Renal carcinoma Melanoma	(33–35)
E2 enzyme	CC0651	Inhibition of tumor cell proliferation	(36)
E3 enzyme	MI - 219	Lung cancer.	(37)
		Chronic myeloid leukemia	(38)
DUB enzyme	Pimozide	Glioblastoma	(39)
	F6/G5	Inducing apoptosis of cancer cells	(40)

E3 Ubiquitin ligase adaptor protein SPOP (Speckle-Typepoz protein) binds the target protein and triggers ubiquitination and proteasome degradation, while BRD4, as a polyubiquitination target, can be ubiquitinated and degraded by SPOP (41–43). It has been reported that SPOP is the most common mutant gene in prostate cancer (44). Further studies have proven that SPOP-mediated BRD4 protein degradation disorder mainly promotes the occurrence of prostate cancer, resists treatment targeting BRD4, and activates the AKT-mTORC1 signaling pathway to regulate tumor cell proliferation, differentiation and apoptosis by increasing the level of BRD4 protein (45). However, studies have shown that SPOP mutations in endometrial cancer promote the accelerated degradation and reduction of BRD4 protein, thus making cancer cells sensitive to BETis (42). The results of BRD4 protein degradation mediated by SPOP mutation in the two cancers are different, and the specific mechanism is not clear, but these findings emphasize that the accumulation of BRD4 protein is the key determinant of the development of BETi resistance as well as a condition for tumor development (41–43). Ubiquitination regulates the stability of BRD4, and mutation or inactivation of its modification enzyme mediates BRD4 to activate certain signaling pathways or recruit oncofactors.

**Figure 2:**
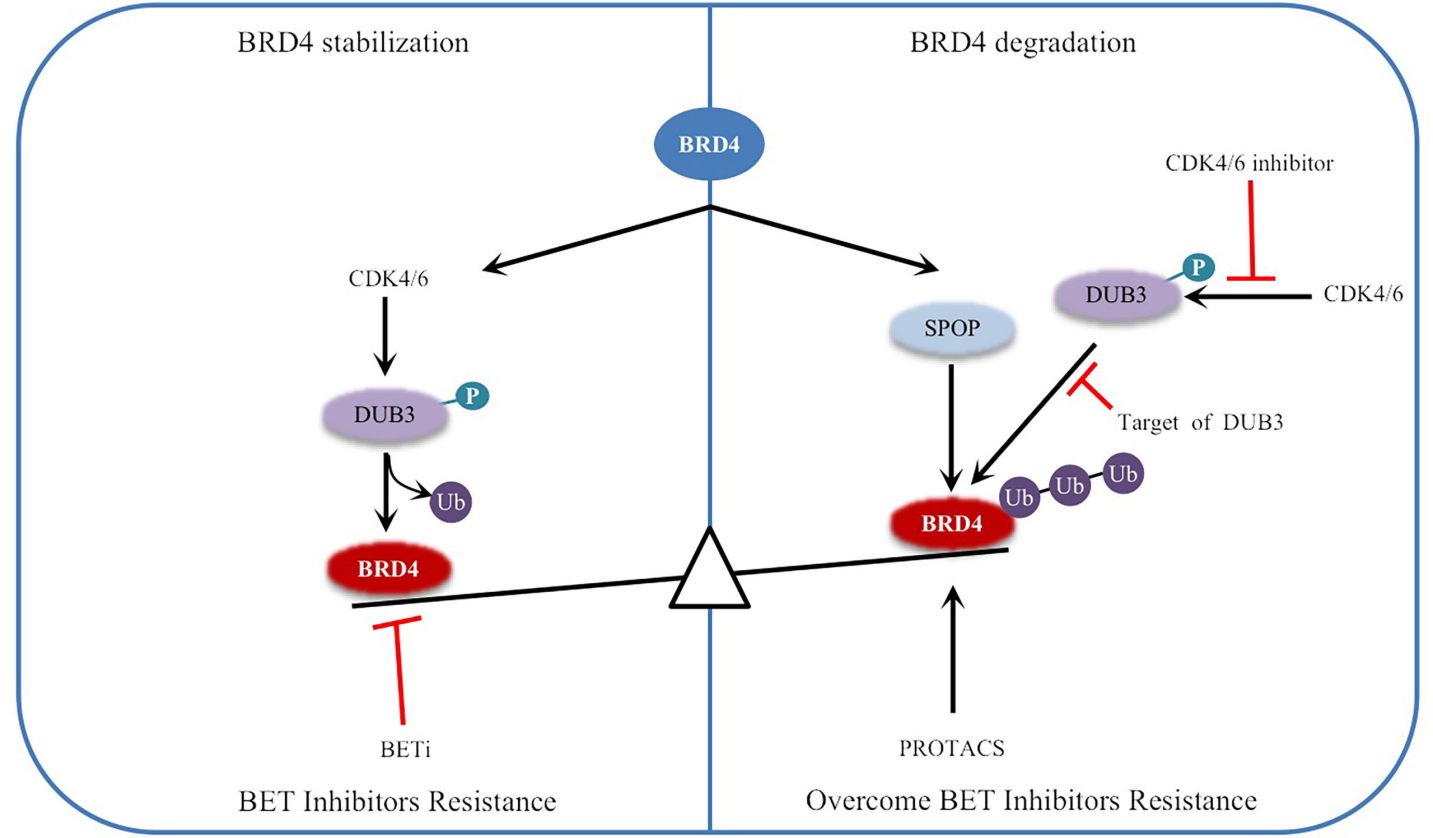
Regulation of BRD4 stability by ubiquitination and deubiquitination: The stability of BRD4 is mediated by SPOP and DUB3. CDK4/6 phosphorylates and activates DUB3, leading to the imbalance between ubiquitination and deubiquitination, and stabilizes BRD4 by antagonizing SPOP mediated ubiquitination, resulting in BETi resistance. Inhibitors of CDK4/6 or targeted inhibition of DUB3 will lead to ubiquitination degradation of BRD4 and overcome BETi resistance. Therefore, PROTACs targeting BRD4 degradation have been developed. Black arrows indicate positive effects. Red perpendicular bars indicate negative effects. Ub , ubiquitin; P, phosphorylation.

DUB3, also known as ubiquitin specific processing protease 17 (USP17), belongs to the DUB/USP family and is highly expressed in a variety of tumors, such as lung, colon, esophageal and cervical cancer (46–48). DUB3 not only stabilizes anti-apoptotic protein MCL1 by deubiquitination, inhibits apoptosis of ovarian cancer cells and drives drug resistance, but also stabilizes oncoproteins (such as CDC25A and Snail) by deubiquitination, promoting breast cancer progression (46, 49), which indicates that inhibition of DUB3 can inhibit the occurrence and invasion of cancer cells. A recent study reported that DUB3 binds to and promotes the deubiquitination and stabilization of BRD4 and protects prostate cancer cells from BETis by promoting BRD4 deubiquitination (43) (**Figure 2**). Other studies have shown that DUB3 is overexpressed in oral squamous cell carcinoma (OSCC) tissues and cell lines and is negatively correlated with patient survival time (50, 51). By inhibiting BRD4 degradation, DUB3 enhanced the expression of EZH2 in OSCC, promoted cell growth and inhibited apoptosis. This indicates that decreasing DUB3 could inhibit OSCC cell proliferation and promote cell apoptosis (50, 51). Overall, DUB3 stabilizes BRD4 through deubiquitination and promotes cancer progression, suggesting that DUB3 may be an effective anticancer target for cancer therapy. Surprisingly, DUB3-mediated deubiquitination and the stability of BRD4 are regulated by CDK4/6, which phosphorylates and activates DUB3 activity (43, 52). CDK4/6 inhibitors(palbociclib)can inhibit DUB3-mediated protein deubiquitination and accelerate BRD4 degradation (43, 52) (**Figure 2**). Therefore, the CDK4/6-DUB3 axis acts as an important regulatory mechanism for cancer metastasis and provides a potential therapeutic approach.

In general, the balance between ubiquitination and deubiquitination is crucial for maintaining the stability of the intracellular environment, and dysregulation may be a carcinogenic factor. Therefore, the combination of targeted modification enzymes or inhibitors of related signaling pathways with BRD4 inhibitors to treat cancer may solve the current problems of poor single-drug toxicity and specificity. However, different tumors may have different key enzymes regulating ubiquitination, which requires further research and exploration. At present, the key enzymes regulating the ubiquitination of BRD4 in prostate cancer and oral squamous cell carcinoma have been reported, indicating that the downregulation of BRD4 expression abundance by targeting the imbalance of the ubiquitination regulation system can effectively control the development of tumors.

## Phosphorylation

**Figure 3:**
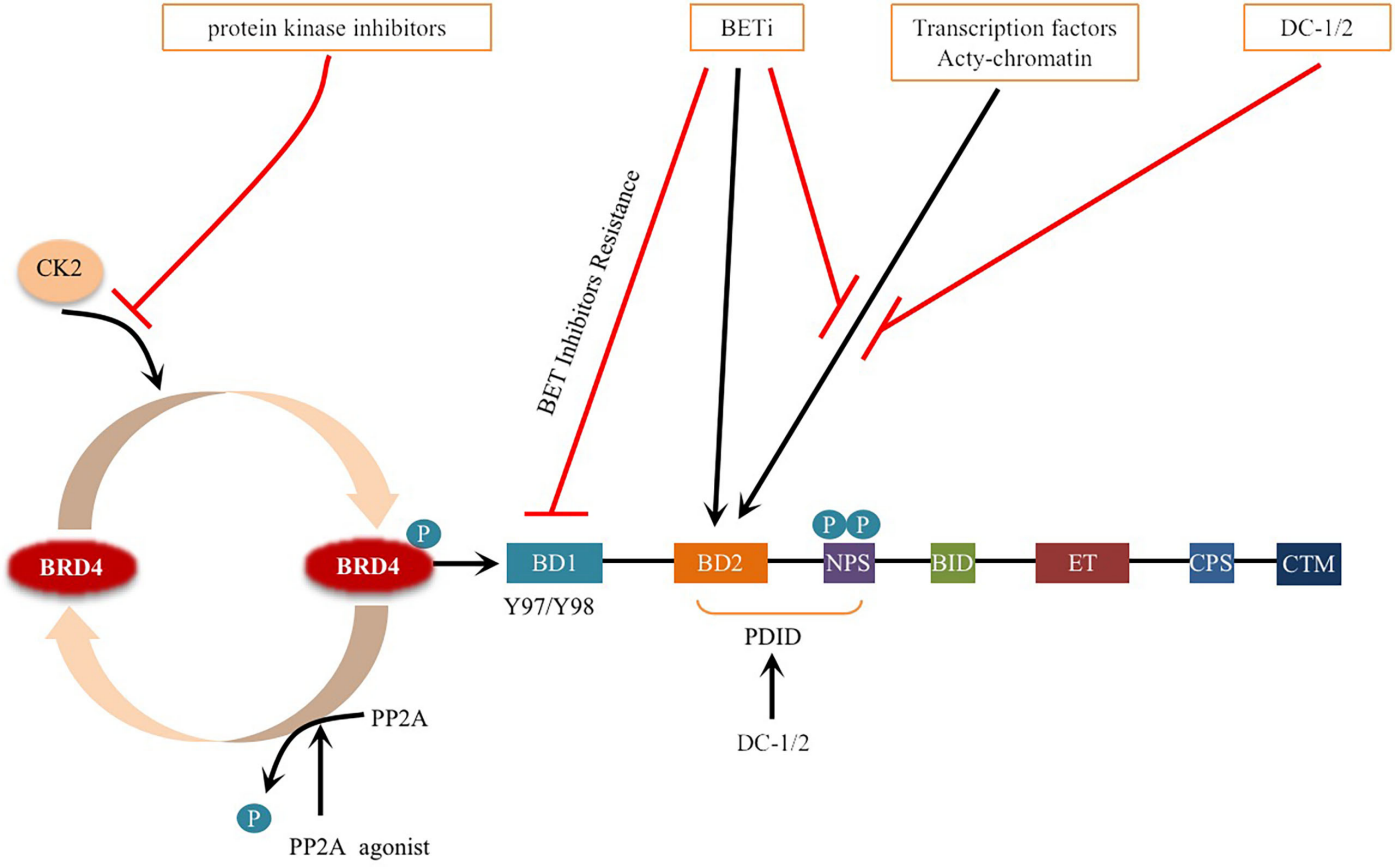
Schematic representation of BRD4 phosphorylation and dephosphorylation: BRD4 intramolecular phosphorylation switch is triggered by CK2 and PP2A, which regulates its function in chromatin targeting, factor recruitment and cancer progression through CK2 phosphorylation and PP2A dephosphorylation. Phosphorylation of Y97/98 at the bromodomain BD1 of BRD4 leads to reduced binding to BETi, resulting in drug resistance. Phosphorylation of (NPS) is the key to BRD4 binding to acetylated chromatin and recruitment of transcription factors to target gene sequences. BETi inhibitors compete with acetylated chromatin, transcription factors to bind to the bromodomain. DC-1/2 was developed for targeting phospho NPS, mediating specific transcription and factor recruitment. Protein kinase inhibitors inhibit the phosphorylation of BRD4. Small molecule activators of PP2A enzyme activity promote the dephosphorylation of BRD4 and prevent BRD4 from binding to acetylated nucleosomes and possibly acetylated transcription factors. Black arrows indicate positive effects. Red perpendicular bars indicate negative effects.

Phosphorylation refers to the attachment of a phosphorylated group to a protein, primarily to serine, threonine and tyrosine, which is catalyzed by kinases (18, 53). Dephosphorylation refers to the removal of phosphate groups, and this process is regulated by protein phosphatases (18). In human cells, 518 protein kinases have been reported to phosphorylate, while approximately 226 protein phosphatases remove phosphate groups (54). In recent years, many studies have revealed the role of dysregulated protein phosphorylation in cancer manifestations, which may lead to tumorigenesis when dysregulated (55). Phosphorylation and dephosphorylation of BRD4 are regulated by a balance between casein kinase II (CK2) and protein phosphatase 2A (PP2A), which regulates its function in chromatin targeting, factor recruitment, and cancer progression (56, 57)(**Figure 3**).

BRD4 has multiple highly conserved common CK2 phosphorylation sites located in two main clusters: one is the downstream N-terminal phosphorylation site (NPS) of BD2, and the other is the C-terminal phosphorylation site (CPS) (57, 58). NPS is the key to BRD4 binding to acetylated chromatin and recruitment of transcription factors to target gene sequences, and it mediates the binding of BD2 to acetylated chromatin through phosphorylation regulation (52, 56, 59). Recent studies have reported that the stability and nuclear localization of BRD4 increase with the phosphorylation of CK2 (57). In addition, BRD4 is hyperphosphorylated in cancer, and this hyperphosphorylation may be the general mechanism supporting its oncogenic activity (57, 60, 61).

In colorectal cancer (CRC), interleukin-6/8 (IL-6/8-JAK2), the matrix signal activated by cancer-associated fibroblasts (CAFs), induces phosphorylation of BRD4 at tyrosine 97/98 (Y97/98), which is stable due to its interaction with the deubiquitinase UCHL3 (62). BRD4 phosphorylation at the Y97/98 site promoted increased binding to chromatin but decreased binding to BETi, resulting in resistance to BETi (**Figure 3**) (62). Further research shows that under the stimulation of inflammatory signals in the tumor microenvironment, the phosphorylation of BRD4 at Y97/98 promotes the interaction with STAT3 and regulates carcinogenic enhancers such as MYC, CXCL1 and CXCL2 to induce chromatin remodeling and promote the transcriptional program (62). These findings suggest that inhibition of the IL6/IL8-JAK2 signaling pathway can eliminate the phosphorylation of BRD4 and sensitization of BETis. This provides a theoretical basis for more effective treatment of CRC.

In the JQ1-resistant lung adenocarcinoma (LAC) cell line, the phosphorylation of BRD4 was increased, and CK2 was identified as its kinase (63). This suggests that CK2 phosphorylation of BRD4 may be related to the JQ1-resistant lung adenocarcinoma (LAC) cell line (63). Further studies showed that JQ1 combined with CK2 inhibitor (CX-4945) could effectively induce the death of lung adenocarcinoma cells and determined the CK2 phosphorylation of BRD4 as a potential target to overcome this cancer resistance (63, 64).

Unlike CK2, which phosphorylates BRD4, cyclin-dependent kinase 9 (CDK9) binds BRD4 through its C-terminal domain (CTM) (61). In addition, since P-TEFb is a heterodimer of CDK9 and cyclin T1 (CCNT1), CDK9 phosphorylation of BRD4 increases with the recruitment of P-TEFb by BRD4 (61, 65). In midline carcinoma of NUT (NMC), BRD4 is hyperphosphorylated, and CDK9 was identified as the potential kinase that mediates BRD4 hyperphosphorylation (61). Blocking the hyperphosphorylation of BRD4 results in the inhibition of downstream BRD4 oncogenes and termination of cell transformation; therefore, compounds that block BRD4 hyperphosphorylation may be an effective therapeutic strategy for NMC (61). At present, many studies have shown that the CDK9 inhibitor flavopiridol (FP) can kill the NUT cell line (66, 67). Therefore, the combination of FP and BETi may have a synergistic effect in the treatment of NMC patients.

A recent study showed that BRD4 was phosphorylated by cyclin-dependent kinase 9 (CDK1) during mitosis and identified four phosphorylation sites, T249, S1045, S1117 and S1126 (68). This phosphorylation can lead to the resistance of cancer cells to BETis. In BETi-resistant cancer cells, CDK1 upregulation and BRD4 hyperphosphorylation were observed, including triple-negative breast cancer (TNBC) (68). Further studies have shown that the combined inhibition of CDK1 and BRD4 can overcome the resistance of cancer to BETis to more effectively kill cancer cells related to BRD4-dependent survival (68).

Based on the above discussion, posttranslational modification of BRD4 phosphorylation is closely related to tumor development and BETi resistance. Although the mechanisms regulating p-BRD4 levels may vary by cancer type, targeting this critical event may provide a feasible treatment for these cancers.

Interestingly, BRD4 is an atypical kinase with eight regions with homology to kinase subdomain motifs scattered across its N-terminal region that can phosphorylate serine 2 in the carboxyl terminal region of RNA polymerase II and transcription factors to regulate the transcription process (69, 70). BRD4 can also phosphorylate myc, leading to its ubiquitination and degradation (69). According to the current research results, we have to ask the following questions: what type of relationship exists between BRD4 phosphorylated by relevant kinases and BRD4 that phosphorylates other factors as atypical kinases, do the two regulate each other and what is the significance of kinase inhibitors inhibiting the atypical kinase activity of BRD4 in the future treatment of BRD4 related cancer? These questions are poorly understood and should be further studied in the future.

Phosphorylation modification and dephosphorylation are in dynamic balance. There are differences in specific functions and modification processes, which are not simple additions and subtractions (71). PP2A, a key enzyme that regulates the dephosphorylation of BRD4, plays an important role in many cellular functions (72). The decreased activity of PP2A leads to the hyperphosphorylation of BRD4 (73). Since the phosphorylation of NPS is necessary for intramolecular contact with BD1 and the intermolecular interaction between BRD4 and some transcription factors, BD2 is masked by NPS when dephosphorylated (56). NPS also functionally damages the chromatin binding activity of BD1 by regulating the BD2-BD1 interaction, which prevents BRD4 from binding to acetylated nucleosomes and possibly acetylated transcription factors (56) (**Figure 3**). Inactivation of the PP2A phosphatase tumor suppressor gene also occurs frequently in breast cancer (74–76). Hyperphosphorylation of BRD4 caused by downregulation of PP2A can restore BCL2L1/BCL-XL gene transcription, resulting in drug resistance to BRD4 inhibitors (73). Studies have shown that in TNBC, p-BRD4 is increased significantly in BETi-resistant cells, and p-BRD4 has been proven to be more efficient in combination with Mediator Subunit 1 (MED1) (73, 77, 78). MED1, as an estrogen receptor (ER) coactivator, mediates breast cancer metastasis and treatment resistance (79). The p-BRD4 and MED1 complex can reactivate Myc expression, leading to BETi resistance, which provides an alternative method to reactivate the anticancer target of BRD4 (73, 77). These studies suggest that the PP2A/BRD4 axis, as a novel molecular target, has potential clinical and therapeutic value in overcoming resistance to BRD4-inhibitor-based therapies (73, 77).

## Hydroxylation

Proline hydroxylation (Hyp) is a key oxygen-sensitive posttranslational modification that is irreversible and catalyzed by proline hydroxylase with oxygen as the substrate and iron, α-ketoglutarate, and ascorbic acid as cofactors (80–84). These modifications affect the structure, activity and characteristics of protein interactions within cells and play a key role in cancer development and disease progression (84, 85). Proline hydroxylase domain (PHD) protein is one of the main enzyme families regulating proline hydroxylation in cells, including three main proteins, PHD-1, PHD-2, and PHD-3 (86, 87). BRD4 is a proline hydroxylated substrate in cancer cells, PHD2 is a key regulatory enzyme for the proline hydroxylation of BRD4, and the hydroxylation site (P536) on BRD4 is located at the junction between the phosphorylation-rich NPS domain and the lysine-rich BID domain (88). BRD4 proline hydroxylation not only regulates the interaction between BRD4 and specific binding proteins, such as CDK9, CCNT1, but also affects BRD4-mediated gene transcription by recruiting P-TEFb to the promoter and activating RNA polymerase II (RNAPII)-mediated transcriptional activity (88). In general, inhibition of site-specific proline hydroxylation can reduce the interaction between BRD4 and other proteins and BRD4-mediated transcriptional activation (88). The PHD2-BRD4 regulatory axis is an important functional pathway for BRD4-dependent gene activation and cell proliferation (88).

PHD2 was significantly overexpressed in AML patients, which shows that PHD2-mediated proline hydroxylation is a potential oncogenic pathway in AML, driving BRD4-mediated gene expression and cancer cell proliferation (88). In addition, studies have shown that the inhibition of proline hydroxylase activity significantly reduced the abundance of proline hydroxylase on BRD4 and the proliferation of leukemia cells (89). In general, the carcinogenic proline hydroxylation-dependent pathway may become a new target for the development of leukemia treatment strategies.

## Other Modifications

Lysine acetylation, like phosphorylation, is a reversible posttranslational modification that is regulated by lysine acetyltransferase (KAT) and lysine deacetylation enzyme (KDAC) (90). Most of the research focuses on histone acetylation; however, in recent years, nonhistone acetylation has gradually become a hot topic (91, 92). PCAF (P300/CBP-associated factor) is a member of the GCN5 (general control nonderepressible 5)-related N-acetyltransferase family protein acetyltransferases, which not only acetylates histones to promote gene transcription but also acetylates nonhistones to directly promote its transcriptional activity and is involved in coordinating many carcinogenic and tumor suppressive processes, such as cell cycle progression, DNA damage reaction and apoptosis (92–94).

In LAC, BRD4 interacts with the transcription factor intestine-specific homeobox (ISX) through its BD2 bromodomain to form the ISX-BRD4 complex (92). It is worth noting that PCAF-mediated ISX acetylation further enhances the ISX-BRD4 interaction by recruiting BRD4, mediates EMT signaling and regulates tumorigenesis and metastasis, further research demonstrated that the acetylation of BRD4 lysine residue 332 (K332) induced by PCAF is very important for the EMT characteristics induced by the ISX-BRD4 complex, which mediates cell migration and invasion by regulating the expression of Twist and Snail transcription factors induced by the expression of the ISX-BRD4 complex (92). Therefore, acetylated BRD4 plays an important role in the metastasis and invasion of LAC, highlighting its potential as a therapeutic target (92). Whether inhibiting the acetylation of BRD4 combined with BETi can increase the efficacy in patients with LAC requires much research and clinical trials.

At present, research mainly focuses on BRD4 as an epigenetic reader that recognizes and binds to acetylated histones to regulate related gene transcription (10). However, the effect of BRD4 acetylation on its function remains to be further studied.

Lysine methylation, a key, dynamic posttranslational modification that regulates protein stability and function, is regulated by lysine methyltransferase and lysine demethylase (95–98). The abnormal expression of methyltransferase in many tumor types has been proven to be related to tumorigenesis and development, which has become the focus of anticancer research (98–100). These enzymes are now considered potential therapeutic targets, and they may be used as potential anticancer drugs in the clinic (100, 101).

**Figure 4:**
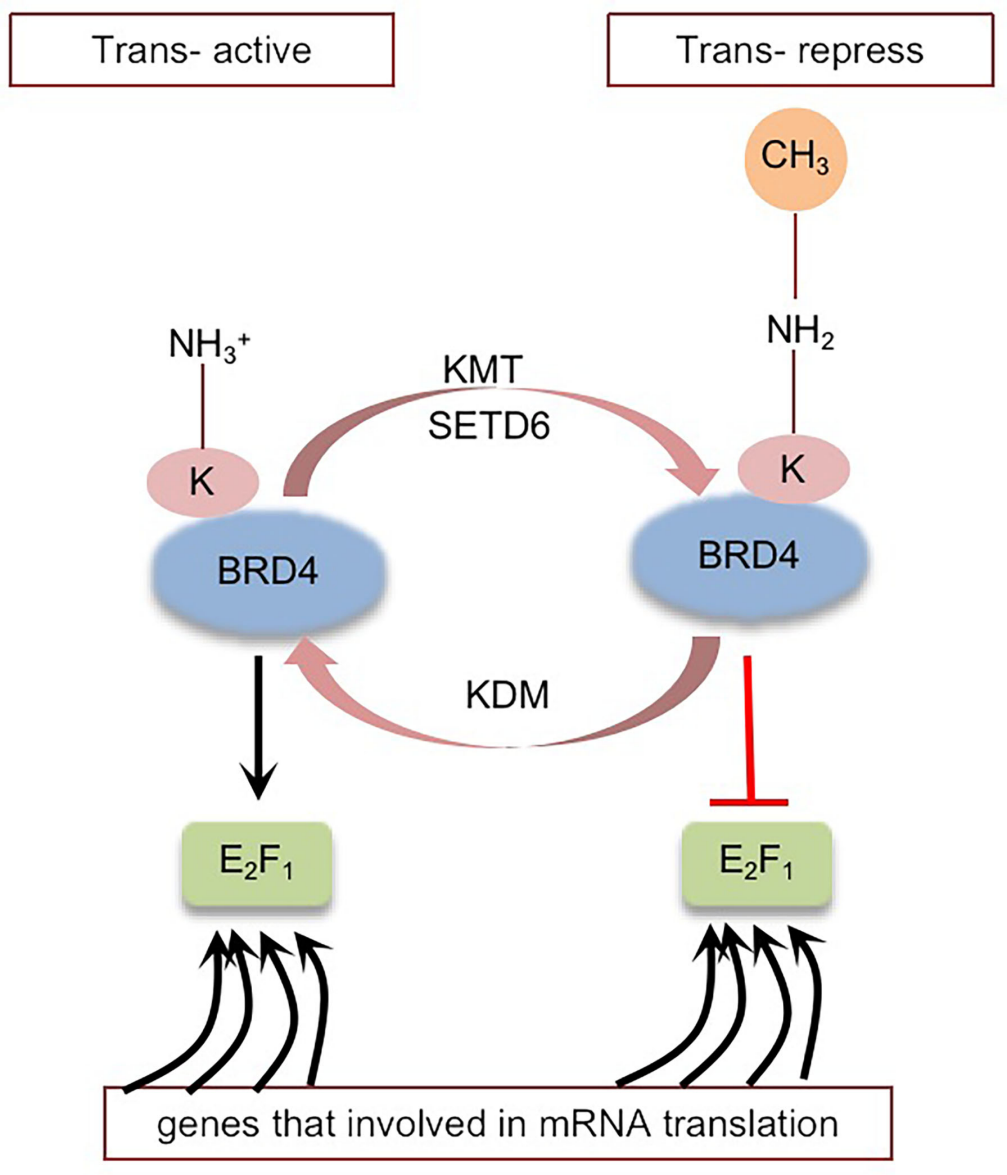
Schematic representation of BRD4 methylation: BRD4 methylation mediated by KMT (SETD6) inhibits the selective recruitment of transcription factor E2F1 to translation related target genes, resulting in global translation inhibition. Black arrows indicate positive effects. Red perpendicular bars indicate negative effects. Tans-active, Translation active; Trans-repress, Translation repress.

BRD4 was methylated by lysine methyltransferase SETD6 on lysine-99 (K99), and further studies proved that the methylation of BRD4 at K99 inhibited the selective recruitment of translation-related target genes by transcription factor E2F1, resulting in overall translation inhibition (102) (**Figure 4**). That is, SETD6, as a molecular switch, determines the methylation state of BRD4, thus affecting the recruitment of E2F1 to genes involved in mRNA translation. The molecular mechanism of BRD4 methylation may provide a new direction for the development of therapeutic applications (95, 102). Whether there are other sites on BRD4 that can be methylated remains to be explored.

There are one other ubiquitin-like proteins, Small ubiquitin-related modifiers (SUMOs), which are involved in a variety of cellular processes, and sumoylation disorders have been implicated in the pathogenesis of human disease (103–106). In recent years, more and more attention has been paid to the role of SUMOylation in protein modification, but there is no clear research report on the SUMOylation of BRD4. We are looking forward to explore whether there is SUMOylation of BRD4. Interestingly, studies have shown that phosphorylation of Yy1 (YY1 transcription factor) in neuronal cells can bind to Senp1 (SUMO-Specific Protease 1) and recruit BRD4, which in turn binds with Senp1, therefore opening the transcription of Senp1, a major de-sumoylase whose upregulation inhibits SUMOylation of proteins in neuronal cells (107). This suggests that in neuronal cells, BRD4 cooperates with Yy1 to inhibit SUMOylation of the protein (107). In tumor cells, whether BRD4 can also regulate the SUMOylation level of the protein by regulating the expression of Senp1, this will be a direction worth exploring.

## Targeting BRD4 Modification Pathway for Disease Treatment

As we discussed above, PTMs of BRD4 can affect BRD4 function in many ways, and now there are some achievements in targeting BRD4 modification in cancer therapy. In recent years, the degradation of BRD4 protein has emerged as a powerful anticancer therapy strategy and could enable this type of treatment called protein proteolysis-targeting chimeras (PROTACs) (108, 109), which consists of three components: the E3 ligase recruiter, link chain, and ligand (mostly small-molecule inhibitor) of the protein of interest (POI) (108–110). PROTACs can induce the ubiquitination and degradation of POI by recruiting E3 ligase to POI in a proteasome-dependent manner, and E3 ligase remains at the heart of the process (111). In this context, the small molecule inhibitor JQ1 has become a versatile and powerful ligand for the development of PROTACs. In recent years, some degraders have been developed for the degradation of BRD4, such as A1874, ARV-825, dBET1, and ARV-771 (112, 113). BRD4 is degraded by PROTACs to overcome BETi resistance caused by BRD4 stability (114, 115) (**Figure 2**). It has been confirmed that PROTACs can effectively induce the degradation of BRD4 and are more effective than corresponding BRD4 inhibitors in inhibiting the growth of tumor cells and promoting cell apoptosis (116, 117).

However, PROTACs, similar to BETis, lack the specificity, selectivity and targets to degrade and inhibit BRD4 in cancer cells and normal cells, which may affect the function of BRD4 in normal cells, leading to unexpected consequences. Therefore, further research on selective and specific targeting drugs is necessary.

Studies have shown that dual BRD4 and cyclin-dependent kinase inhibitors have synergistic effects in cancer treatment (118), for example, the combination of a BRD4 inhibitor (JQ1) and a CDK7 inhibitor (THZ1) had a synergistic effect on head and neck squamous cell carcinoma (HNSCC) (119). In addition, protein and protein interaction (PPI) inhibitors that block the interaction between BRD4 and other proteins are also being developed. For example, N-substituted oligoglycine DC-1 and DC-2, isolated from a combinatorial compound library of approximately 38500 peptides, can specifically bind to phosphorylated PDID/NPS in BRD4, providing an effective strategy to inhibit the phosphorylation-dependent gene-specific function and factor-regulated transcription program of BRD4 (120, 121) (**Figure 3**). Targeting the surface on which phospho-NPS interacts with specific transcription factors highlights the selective suppression of BRD4-regulated transcription processes, unlike BETis, which nonselectively shut down the universal chromatin binding activity of BRD4 (56, 121). Currently, dual kinase/BET inhibitors are in clinical trials for leukemia (122, 123), and the combination of JQ1 and a CK2 inhibitor (CX-4945) can improve the efficacy in the treatment of patients with acute lymphoblastic leukemia (ALL) (124). Obviously, targeting phosphorylation pathways provide optimistic prospects for the development of a new generation of antitumor drugs. In addition, PP2A agonists such as SMAP and phenothiazine have been developed for cancer treatment (72, 73) (**Figure 3**).

At present, not only our understanding of the types of BRD4 modifications is limited, but also the development of modified BRD4-targeted drugs is difficult. Further research in this area may show clinical prospects. We look forward to the development and clinical application of more drugs targeting BRD4 modification.

## Conclusion

BRD4 disorders have been implicated in the pathogenesis of many cancers, such as AML, multiple myeloma, Burkitt’s lymphoma, diffuse large B-cell lymphoma, and breast, colon, and ovarian cancers (117, 125–130). BETis have been developed for BRD4-targeted inhibition; however, to date, their efficacy as a single drug is limited. As with most established cancer therapies, primary and acquired tumor resistance may limit the clinical application of the drug and reduce its efficiency. In addition, BETis can inhibit BRD4 in cancer cells and normal cells, which may have an adverse effect on the function of BRD4 in normal cells. Studies have shown that the cross-linking of BRD4 function and kinase signaling is one of the main mechanisms of BETi drug resistance (43, 45, 73, 131). Therefore, further study on the function and specific regulatory mechanism of BRD4 may provide a new idea for the treatment of targeted BRD4 in the future, which is likely to solve the problems of BETi resistance and toxic side effects of monotherapy and improve antitumor activity. Here, we review several specific PTMs of BRD4 and show how these modifications can modulate BRD4 to produce different results, pointing to potential targets that could not only help design more effective anticancer treatment strategies but also selectively turn off the carcinogenic activity of BRD4 in cancer cells. At present, BETis combined with other drugs (such as targeted drugs and/or other epigenetic drugs) have been used in preclinical and clinical trials to treat various cancers. In addition, more efficient PROTACs are also being developed to degrade BRD4. We expect specific and multitarget BETis to continue to develop rapidly as candidate drugs for cancer treatment. However, to date, the understanding of BRD4 modification sites, modification types and related enzymes is limited, and further exploration of various modification types of enzymes and associated sites will help to better understand the function, mechanisms, regulatory and therapeutic applications of BRD4. Although PTM-based therapies are promising for future cancer treatment, the following issues still need to be addressed: will different modifications of BRD4 regulate each other or even compete for the same modification site and what is the significance of the interaction network among different modifications in tumorigenesis, development and treatment? Little is known about these areas, and future research should guide further testing of these assumptions.

## Author Contributions

DC and NL conceived and designed the review. NL, XT, RL, YY, and YZ wrote, edited and revised the manuscript. All authors have read and approved the final manuscript.

## Funding

The present study was funded by The National Natural Science Foundation of China (grant no. 81572956), The Jiangsu Provincial Science and Technology Supporting Program (grant no. BE2017696), The Medical Innovation Team of Jiangsu Province (grant no. CXTDC2016009).

## Conflict of Interest

The authors declare that the research was conducted in the absence of any commercial or financial relationships that could be construed as a potential conflict of interest.

## Publisher’s Note

All claims expressed in this article are solely those of the authors and do not necessarily represent those of their affiliated organizations, or those of the publisher, the editors and the reviewers. Any product that may be evaluated in this article, or claim that may be made by its manufacturer, is not guaranteed or endorsed by the publisher.
